# Nature’s Role in Outdoor Therapies: An Umbrella Review

**DOI:** 10.3390/ijerph18105117

**Published:** 2021-05-12

**Authors:** Nevin J. Harper, Carina R. Fernee, Leiv E. Gabrielsen

**Affiliations:** 1 Faculty of Human & Social Development, University of Victoria, Victoria, BC V8W 2Y2, Canada; 2 Sørlandet Sykehus, 4604 Kristiansand, Norway; Carina.Fernee@sshf.no (C.R.F.); Leiv.Einar.Gabrielsen@sshf.no (L.E.G.)

**Keywords:** umbrella review, nature, therapy, outdoor therapies, health

## Abstract

*Objective:* To report on the role of nature in outdoor therapies through review and summary of existing systematic and meta-analytic reviews in an effort to articulate a theoretical framework for practice. *Materials and methods:* An umbrella review was conducted following systematic protocols PRISMA guidelines. *Results:* Fourteen studies met the inclusion criteria and represented five self-identified approaches: nature-based therapies, forest therapy, horticultural therapy, wilderness therapy, and adventure therapy. Clear and comprehensive descriptions of theory, program structure, and activity details with causal links to outcomes were mostly absent. *Conclusions:* A rigorous and determined program of research is required in order to explicit in-depth theories of change in outdoor therapies. Conversely, or maybe concurrently, a holistic theory of integrated relatedness may be developed as a parallel expression of support for nature in therapy while the explanatory science catches up.

## 1. Introduction

Understanding therapeutic values of human contact with nature is a topic of growing interest across health promotion and treatment fields [1]. A common narrative in developed nations is that urbanized and technology-driven lifestyles have diminished healthy human relationships with natural environments leading to a range of health issues and reduced wellbeing [2]. While long-acknowledged as practices across cultures, outdoor nature-based therapeutic interventions have grown significantly in number and type in recent years [3]. Outcomes research has supported outdoor therapeutic approaches as improving the lives and wellbeing of those experiencing mental health issues [4,5] as well as for general health promotion [6]. While an evidence base is present supporting exposure to nature and green spaces as ‘pathways’ toward comprehensive health benefits [7], a lack of comprehensive theoretical and conceptual articulation exists specifically for nature’s contribution or role in psychotherapy and mental health outcomes, leaving outdoor therapies without an explicit theory of change for their application as a clinical practice.

Outdoor therapies are intentional therapeutic processes that are (1) place-based, (2) feature active bodily engagement, and (3) recognize nature-human kinship [8]. Significant variety exists in practice, from walk and talk therapy [9], to expedition-based wilderness therapy [10,11], to garden and animal-assisted therapies [12]. These approaches have shown improvement across a wide range of social, emotional, physical, physiological, and psychological outcomes and populations [13,14,15]. While numerous approaches exist, all purporting contact with nature as a common essential component, theoretical development articulating mechanisms of change and processes contributing to the therapeutic outcomes are lacking [8,16,17]. We postulate this knowledge gap exists due to the diversity of therapeutic approaches and subsequent study designs [18,19].

Understanding and describing mediators (relationships between intervention and outcomes), moderators (characteristics that influence direction or magnitude between the intervention and outcomes), and mechanisms of change (the steps or effects—described with specificity—that produce change) in therapy is complicated. A concerted and systematic effort is required to understand how and why change is taking place in practices within outdoor therapies [20,21]. An umbrella review [22] was chosen to provide an overview of the guiding theoretical frameworks drawn upon in the outdoor therapy literature through an overall examination of the body of systematic and analytic reviews. Papatheodorou [23] states “The advantage of this method is that we may be able to collectively evaluate the state of the evidence in broad categories of research, which may make more sense in clinical practice than evaluating [them] one by one.” (p. 543).

### The Present Study

The overall objective of this umbrella review is to report on the role of nature in outdoor therapies through a summative review of existing published systematic reviews and meta-analyses. Our specific objectives were to identify: (1) theories explaining nature’s role in outdoor therapies, (2) nature’s mechanisms of change in outdoor therapies, and (3) recommendations for practice and research within the field of outdoor therapies.

## 2. Materials and Methods

An umbrella review was conducted following systematic protocols set out by Aromataris et al. [22] and following PRISMA guidelines.

### 2.1. Search Strategy

Studies were identified by systematically searching electronic databases selected to represent three distinct fields of study related to our knowledge of the outdoor therapies literature: PsychINFO (psychology), Medline (medicine), and Academic Search Premier (education and multi-disciplinary). Each database provided adequate control over filters to utilize consistent and strict search terms, which would return meaningful results. Search terms were proposed by the first author using PsycINFO thesaurus to identify possible key word for studies investigating outdoor therapies. Search terms were then agreed upon by all authors through exploratory searches, appraisals of other review findings, reduction of terms through testing (e.g., to increase specificity of findings without losing ideal reviews), and open dialog prior to running the database search. Further, consistent search criteria details included that studies had to be published in English and were peer-reviewed. No publication date restrictions were applied and the final comprehensive search was conducted 15 August 2020. Table 1 displays search terms and results by database.

### 2.2. Selection Criteria

All studies were imported into Covidence software [24] to manage documents and guide the inclusion/exclusion and voting procedures of the umbrella review. Covidence facilitates review studies with multiple researchers, manages criteria and inclusion/exclusion choices throughout review stages, maintains confidentiality between researchers when reviewing studies, and provides researchers with an audit trail of decisions and outcomes [25]. Specific eligibility criteria were established utilizing a PICOS modified statement (see Table 2). Further detailed questions were developed and agreed upon for screening, allowing all three researchers to ‘vote’ on inclusion/exclusion of papers with common language and understandings. Two rounds of screening (i.e., title/abstract & full text) occurred with two researchers completing independent and confidential reviews concurrently in each round. Conflicts were resolved by the third researcher and then discussed openly to ensure consensus for the decision was achieved.

**Figure 1:**
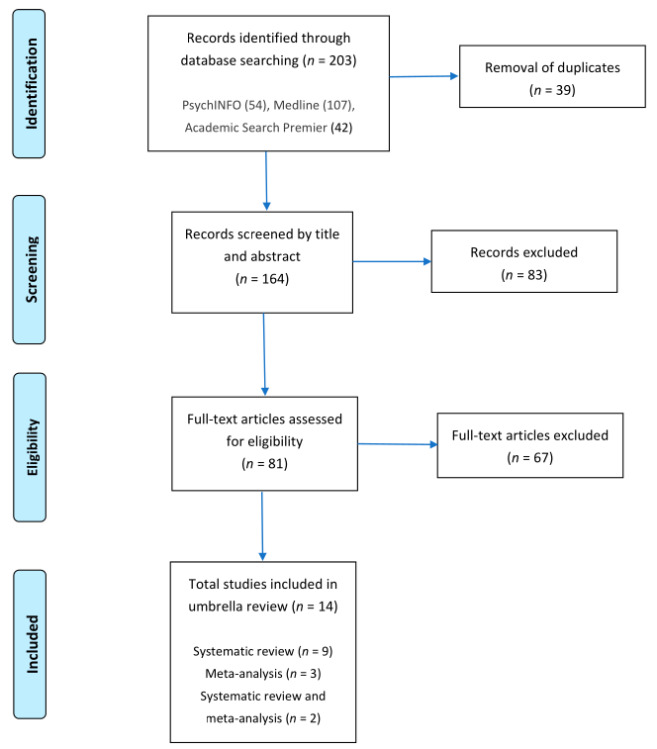
PRISMA flow chart of the study selection and inclusion process.

The first screening was completed (by NJH & LEG) at the level of title and abstract only and resulted in an exclusion of 81 studies for not meeting the PICOS criteria. At this point in the study, two umbrella reviews in specific outdoor therapies were located in a sperate search; one in animal-assisted interventions [5] and one in equine-assisted interventions [12]. After careful consideration, we chose to remove systematic and meta-analytic reviews of animal-assisted and equine-assisted studies to avoid repeating this recently completed scholarly work, and in recognition that therapeutic relationships in animal-assisted therapies are triangulated with client, therapist and another interactive species (i.e., dog, horse, etc.) while the remaining approaches utilize the environment (i.e., nature) as the setting for therapy—the focal point of our study. A second round of review was completed (by NJH & CRF) at the level of full text and saw another 67 studies excluded for not meeting PICOS inclusion criteria (i.e., wrong intervention such as a health promotion focus without a therapeutic component, or wrong study design such as a scoping review). The PRISMA flow chart [26] (Figure 1) below displays the selection process resulting in the final 14 studies to be included in the present review.

### 2.3. Data Extraction

Processes and criteria for data extraction, guided by our PICOS statement, were agreed upon a priori by all researchers. Two researchers (NJH & CRF) independently completed extraction utilizing the following categories: populations, context, theory, mechanisms of change, recommendations for practice, and recommendations for research. Conflicts on data extractions were resolved through dialog and inclusion of a third researcher (LEG).

## 3. Results

Table 3 outlines the characteristics of the included 14 studies in alphabetical order. A brief description of the studies follows to provide further context and details regarding populations served, the types of outdoor therapy interventions, treatment goals, and study design.

### 3.1. Study Descriptions

Ten of the studies included youth participants, eleven included adults (one specific to 60+ year olds), and only two studies included children. A range of contexts were identified across the 14 studies including studies assessing mental and physical health, specific diagnosed health issues, and wellbeing.

Of the 14 studies in this review, seven were located in the discipline of public health [27,29,30,31,35,36,38], two in medicine [33,37], two in child, youth, and family studies [28,32], one in mental health and addictions [34], one in psychology [18], and one in nursing [39]. The studies were organized into five categories based on their self-identified approaches: nature-based therapies [27,29,30,31,36], forest therapy [34,35,38], horticultural therapy [33,37,39], wilderness therapy [28,32], and adventure therapy [18]. Nature-based therapy is defined as “an intervention with the aim to treat, hasten recovery, and/or rehabilitate patients with a disease or ill health, with the fundamental principle that the therapy involves plants, natural materials, and/or outdoor environment[s]” [27] (p. 372). Forest therapy (or forest bathing or shinrin-yoku) is defined as a healing practice in which “people immerse themselves in nature, while mindfully paying attention to their senses” [34] (p. 1). Horticultural therapy is defined as “the engagement of a person in garden-related activities, facilitated by a trained therapist, to achieve a specific treatment goal” (p. 931, American Horticultural Therapy Association, cited in [33]). Wilderness therapy is defined as a form of residential treatment, primarily for adolescents, and may include expedition trekking or base camp models with a focus on immersion in wilderness locations [28]. Adventure therapy is defined as programs or services that “utilise outdoor activities and experiential learning exercises to help participants to deal with their psychological problems” [18] (p. 28).

### 3.2. Quality Assessment

Studies were evaluated for quality using the Joanna Briggs Institute’s (JBI) [40] critical appraisal checklist for systematic reviews and research synthesis. The checklist is comprised of 11 questions used to assess the methodological quality and comprehensiveness of systematic and meta-analytic review studies. Each question could elicit the responses yes, no, unclear, or not applicable and then receive an overall appraisal to include, exclude or to seek further information. Two reviewers (NJH & CRF) completed the quality appraisal and were blinded to each other’s assessments until compared. If consensus was not apparent, open dialog led to resolution, with the option to bring the third reviewer (LEG) into the process as needed.

Critical appraisal of the studies resulted in maintaining the inclusion of all 14 papers. Most papers included necessary elements required of a systematic review as suggested by the JBI checklist. Three areas of weakness in about a third of the papers was a lack of recommendations for research, recommendations for practice, and a thorough explanation of how data extraction errors may have been minimized. It is worth reminding readers that a quality rating for an umbrella review is not a quality assessment of the individual studies within each review, but rather how well the systematic reviews and meta-analyses were conducted and reported. Overall, while some weaknesses were present, these papers were consistent in reporting according to protocols established for umbrella reviews [22].

### 3.3. Theory

The primary objective of this review was to identify clear expressions of nature’s role in outdoor therapeutic practices. While included reviews shared some conceptual arguments in their respective literature review sections, we were searching for theories of change reported in the results and discussion sections of each included review. In other words, we were seeking to identify operationalized theoretical frameworks explaining nature’s role in therapy, however we found little.

Direct reference to theories other than allusions to specific activities and therapeutic approaches was limited to Attention Restoration Theory (ART) [41] and Stress Reduction Theory (SRT) [42], both named in Kotera et al.’s [34] recent review of forest therapy and mental health outcomes. ART posits that time spent in compatible natural environments creates the experience of soft fascination (attention that becomes involuntary) and thereby allows for a renewed energy and ability to pay attention—hence, reflecting the theory’s name [41]. SRT suggests that our positive physiological responses to safe natural environments is evolutionary and reduces levels of stress. Being away is another construct of ART and Kamioka et al. [33] identified that being free from the stress of work and experiencing joy was the main reason found in their systematic review of horticultural therapy. Wen et al. [38] described the characteristics and seasonality of the forest interacting with the five senses as the central features of forest therapy in reaching improved mental health outcomes. There is also a reference to Gilbert’s model of affect regulation suggesting evolutionary emotional responses (e.g., soothing) to being in nature [43].

Annerstedt and Währborg [27] suggest that modern society and lifestyles have become overly stressful resulting in ill states of human health, and that health can be better defined by the fit and adjustment between person and environment. It is for this reason, they posit the effects of nature-based therapy have relevance and success. Djernis et al. [31] echo the above in stating that nature as context “may play a significant role in the benefits” of these interventions. Playing on the language of ART they suggest the environments may be “so fascinating that it calls for soft attention, thereby allowing disengagement” (n.p.). This conceptualization suggests nature stimuli hold our attention and reduce the likelihood of our mind wandering.

### 3.4. Mechanisms of Change

“From the included articles it is also difficult to draw any conclusion of what are the underlying explanatory mechanisms behind NAT’s [nature-assisted therapies] functionality and why certain programmes or therapies are more efficient than others. This is often rather vaguely discussed and no determined explanations are given.” [27] (p. 383).

Annerstedt and Währborg [27] set the stage for this section as we were hardly able to identify clear mechanisms of change across all 14 papers. Our search for mechanisms of change was defined by causal connections with empirical grounding, whether social, relational, or psychological. What we found, however, was that clear and comprehensive descriptions of theory, program structure, and activity details with causal links to outcomes were mostly absent. For example, Britton et al. [29] stated “Details about the blue space setting or the natural environment were limited in all studies…” and suggested that “the how and why a particular nature setting was selected would strengthen the interpretation of intervention outcomes” (p. 64).

Kotera et al. [34] also spoke to the lack of research and discussion about nature’s potential role, specifically in relationship to affect regulation. They stated that their review findings are in alignment with models of emotional regulation related to threat, drive, and the calming of the autonomic nervous system. While not identified as a causal mechanism, the authors suggest that facilitated positive experiences in nature “may activate our soothing system, endorsing compassion, safety and connection, protecting our mental health” (p. 20).

A few correlations (postulating possible mechanisms of change) were identified in this review, including the role and impact of the facilitator, a person-in-environment or bodily-felt experience, and the ‘five senses’ approach. The role and potential need of a trained facilitator was discussed as likely linked to processes [33,36] and subsequently the reported outcomes [28]. Djernis et al. [31] found explanations of nature increasing the experiences of memory and thereby extending the longevity of benefits gained. Britton et al. [29] pointed to the characteristics and diversity of natural environments and how humans interact with them. They provide the example of water-based programs where movement and balance are engaged (i.e., vestibular and proprioception systems) and submersion in water which can reduce pain, alter body sensations and provide an equitable experience for someone with physical limitations. Further, they found connections between physical challenges of the wilderness therapy programs on the physical body (i.e., aches and pain) and reports of increased self-efficacy and resilience. Again, these findings do not clearly identify mechanisms of change. Lee et al. [35] shared the common activity of walking in forest therapy as well as the ‘five senses’ approach which appears to be sometimes manualized, but also practiced with great diversity, such as “forest viewing, forest meditation, Qi-Qong, aromatherapy, herbal tea therapy, and craftwork using natural materials” (p. 11). The authors also posited that just being present in nature, or just viewing nature, may not be enough—suggesting that more needs to be known about the dosage, protocols, practitioner influence, and whether or not outcome measures utilized are actually capturing what they intend to.

### 3.5. Brief Summary of Evidence

This primary objective of this review was to identify theories and mechanisms of change guiding outdoor therapies. With scant theory and causal mechanisms identified, there exists a considerable gap in knowledge as to how outcomes in outdoor therapies are achieved. Clinical and practical outcomes reported in each of the 14 papers we reviewed suggest positive benefits from “moderately positive” to “effective.” The reviews of nature-based therapies suggest some improvements in mental health and psycho-social wellbeing [29], psychological states related to emotions and stress reduction, but that the evidence was weak regarding the links to physiological outcomes [31]. Forest therapy reviews showed positive outcomes for mental health, particularly anxiety [35], and physiological improvements such as reducing blood pressure and boosting immunity [38], again failing to show casual links between physiological change and mental health outcomes. Horticultural therapy reviews stated positive outcomes for a range of mental health and behavioral disorders “such as dementia, schizophrenia, depression, and terminal-care for cancer” [33] (p. 942) as well as for “cognitive function, agitation, positive emotion and engagement” [39] (p. 14), again depicting connections but not causal links. Wilderness therapy reviews suggested outcomes measured across a range of social, psychological and behavioral constructs with positive treatment effects in the areas of self-esteem, locus of control, behavioral changes, personal effectiveness, clinical symptomology, and interpersonal skills [28,32]. Bowen and Neill’s [18] adventure therapy meta-analysis was significant in size and scope (197 studies, 2908 effect sizes, 206 unique samples). The authors posited that adventure therapy produces moderate short-term outcomes and that positive gains are maintained over the long-term indicating a robustness of outcome over time [18]. Overall, strong outcomes, but lacking clear indications of mechanisms of change.

In sum, the evidence of treatment outcomes across outdoor therapies is mostly positive. The breadth of interventions were diverse and all were comprised of being place-based/outdoors, with active engagement of bodies-in-environments, and often focused on increasing one’s connection to nature. While populations served and issues addressed varied, the approaches all included direct contact with nature and other species. Still, we were unable to identify clear articulations of nature’s contribution to the specific therapy undertaken. ART and SRT were both presented in an explanatory manner but fall short of explaining outcomes. Descriptive models of change in these therapies is sorely lacking and in need of development, along with clear descriptions of practice for future researchers to design and plan research by. Echoing these needs, Britton et al. [29] stated “a consistent lack of description of setting characteristics or the natural environment as a ‘subject’ was evident across all of the studies” and further highlighted the “need to improve our understanding of complex nature-based interventions for health outcomes” (pp. 64–65).

### 3.6. Recommendations for Practice

Public health authorities are beginning to recognize the value of proximity to, and contact with, natural environments as upstream health promoting interventions for the population on the whole [44]. A term increasingly used in environmental policy and management is “nature-based solutions” [29] (p. 42), where time outdoors and nature-based interventions have become more relevant than ever for overwhelmed individuals, families, schools, and healthcare services during the COVID-19 pandemic [45]. Most included reviews recognized the need to address the immense pressure that earth’s ecosystems face and the growing disconnect and detachment from our natural surroundings [29]. We will echo that and remind readers of the basic ecopsychology principle that intricately links environmental health and human health.

Thus far, theoretical models, on which to base both practice and research, are not concisely defined, nor utilized for testing and implementation. Annerstedt and Währborg’s [27] observations still holds true, when stating that: “we are still not satisfied in aspects of evidence, quality, or causality, what specific natural elements are most beneficial, and to what population with what diagnoses” (p. 385). Britton and colleagues [29] point to the lack of a common language for nature-based providers, researchers and policy-makers, where conceptual ambiguity of central terms such as nature, health, and wellbeing exacerbates a lack of coherence across nature and health research, policy, and practice. Other recommendations include increased local collaboration across practice and research, which can lead to greater engagement and sustainability over time through the integration of nature-based practices into the existing community structures and services.

Unfortunately, while nature + therapy seems to be beneficial, the knowledge base within outdoor therapies to date remains a conditional basis for effect inferences and the heterogeneity within the field makes it premature to formulate specific recommendations for practice at this stage [36]. In order to inform best practice guidelines, more high quality, exploratory, in-depth and theory-generating research is needed as a means to clarify the impact of different types of outdoor therapies for various populations and develop detailed theories of change.

### 3.7. Recommendations for Research

Outdoor therapies present certain core challenges for the conduct of high-quality research, where the complex interaction between individual subjective experience, activities, pedagogies, and places are difficult to operationalize and measure in quantitative terms [36]. The practices are often inscribed in real-life contexts where it is not feasible or desirable to randomize participant allocation, and the aforementioned complexity may compromise the overall transferability and generalizability across the field of outdoor therapies. Moving forward, Mygind and colleagues [36] propose practitioners and researchers alike ought to channel a substantial effort into formulating program theories. Such a theory should identify: (1) Intervention input; activities; outputs; intermediate outcomes; and long-term outcomes; and (2) a theory of change, i.e., description of how and why the desired change is expected, pertaining to the specific type of nature experience and intended health outcome. In such theories of change, not only should the type and characteristics of nature be considered, according to Mygind et al. [36], but also the way the natural environments are activated, including the social and pedagogical processes that it affords more specifically. Such a theory can also guide research into which types of nature, and which qualities of those types of nature, are relatively effective for particular targeted outcomes. In addition to the descriptions of the intervention and the outdoor environment, future studies should also provide clearly defined and specific details with regards to: (3) the individual participants, e.g., age, gender, ethnicity, sociodemographic status, and needs/diagnoses, (4) the group, if applicable, and (5) qualifications and roles of instructors/guides/therapists.

Through this umbrella review, we have elicited a number of recommendations for outdoor therapy research: (1) The use of validated psychometric assessment tools [18,28] and physiological measures, in addition to self-reported questionnaires and observational measures, in order to capture a fuller picture of the therapeutic effects of outdoor therapies [35,37]; (2) identify and measure process factors [28]; (3) provide matched sample results, as well as means, standard deviations and sample sizes for each outcome at each measured time point [18]; (4) follow-up and longitudinal assessments to test longer term effects [34,35]; (5) more studies with clinical samples [35]; (6) increased involvement of participants’ perspectives in the design and delivery of interventions [29]; (7) increased attention to participants’ perceived experience of and attitudes toward particular outdoor environments and activities, as well as their previous engagement, or lack of engagement, with nature [29]; (8) describe any side-effects, adverse events or harmful phenomena, including reasons for withdrawal and non-participation, in outdoor therapy studies [27,33,37]; (9) include cost-effectiveness information [27,33,36]; and finally, (10) utilize randomized control designs when possible and appropriate [28,33].

When it comes to the latter request for randomized controlled designs (RCT), randomization is not always feasible, ethical, or acceptable, acknowledging the difficulty in designing controlled or clinical interventions in outdoor, natural, and/or water settings compared to more controllable environments, typically indoors [29]. When applied, procedures of randomization and blinding must be described. Kotera et al. [34], in their review on forest bathing, found that some RCTs used a cross over design with minimal interval, which violates the accuracy of the results. In circumstances where randomization is not practical or possible, Mygind and colleagues [36] propose that regression discontinuity and comparison time series designs may be feasible alternatives for achieving unbiased estimates of intervention effects. Other types of quasi-experimental designs include dynamic wait-listed designs, in addition to so-called stepped wedge and regression point displacement designs [36]. A potential weakness in the use of RCTs in the context of outdoor therapies is that they tend to focus on outcomes and therefore fail to account for how social and environmental processes influence treatment trajectories [29].

Further areas for research include a request for investigating ethical implications for outdoor therapies and evaluating the impact of nature-based solutions on public health [29]. Kotera et al. [34] also recommend a closer examination of psychological constructs such as nature connectedness and other constructs that are relevant to well-being and the soothing system, such as self-compassion and psychological safety. Wen et al. [38] calls for more interdisciplinary collaboration, particularly between scholars within medicine and forestry when it comes to forest therapy specifically. At present, most studies are based on qualitative or quantitative analysis of evidence-based medical research, lacking basic theoretical insights of forestry. Comprehensive analyses of the forest or natural environment combined with dynamic monitoring of key factors of the therapeutic process are important to comprehend the multi-dimensional health benefits of outdoor therapies and for developing theories of change [38].

Outdoor therapy studies are dominated by the use of self-referred subjects, who might be expected to have an interest in nature and natural environments, which again could cause a potential “nature-positive” bias [30]. Not all experiences are deemed to be positive, yet selection bias could favor those who had more positive experiences [29]. Furthermore, there is a need for contextually sensitive and process-oriented approaches in outdoor therapies research that measure more than “what” worked or did not work well; but also evaluating “how” and “why” success, or indeed failure, happened [29]. The epistemology of classical science and linear understandings are not sensitive enough to the dynamics and complexities of “messy” nature-based interventions. Future research could therefore benefit from operationalizing inter-disciplinary frameworks such as complex systems approaches that assume multi-causality and a non-linear perspective, in order to arrive at more holistic understandings of outdoor therapies in a socioecological context [29].

## 4. Conclusions

Outdoor therapeutic practices may serve as accessible and affordable upstream health promotion with the potential to enhance mental, physical, and social wellbeing for various populations. In general, activities, pedagogies, and types and qualities of the natural environments of the outdoor practices under investigation were not presented in sufficient detail. Furthermore, given that practices are culturally and geographically bounded, descriptions of local conditions should be considered and described carefully in future research. While systematic reviews to date have synthesized the wealth of predominantly correlational literature exploring nature contact and health benefits across highly heterogeneous interventions, the current evidence is both diverse and dispersed, leaving us with simplified conclusions, reduced interpretational value, and potentially inappropriate recommendations [36].

To the best of our knowledge, this is the first umbrella review of outdoor therapies: horticultural, nature-based, forest, wilderness, and adventure therapies combined. As further systematic and meta-analytic reviews are completed in each of these areas, a specific umbrella review will be of benefit to further capture the volume and quality of the evidence within each approach. We hope that the progression of research in these specific areas of practice will contribute towards further defining theory and mechanisms of change. Two recent studies have utilized a realist methodology to attempt to capture the complexity and make theoretical progress within the field of outdoor therapies. Fernee et al. [10] set out to unpack the treatment process of wilderness therapy for clinical youth populations, while Masterton et al. [46] explored greenspace interventions for mental health in clinical and non-clinical populations. Masterton and colleagues put forth detailed program theories, as requested by Mygind et al. [36] above, which can serve as hypotheses to be tested and refined in future studies.

We recognize growing and now heightened interest (i.e., outdoor activity for health during the pandemic) in these approaches as active practitioners and researchers. Guidelines have recently been put forth for the use of talking therapy outdoors from the British Psychological Society and the statement for good practice for outdoor mental health intervention and outdoor therapy from the Institute of Outdoor Learning [47]. Other publications of great relevance for the practice of nature-based solutions and outdoor therapies are the Oxford textbook on nature and public health [44], the international handbook of forest therapy [48], and the first introductory text of outdoor therapies [19].

In closing, we found the causal approach (theory—mechanisms—outcomes) to our review unfruitful, and while echoing others’ calls for more specific research, we also conclude that it may be difficult to find what we seek: nature’s role in outdoor therapies. Outdoor therapies take place in complex social and environmental contexts with almost infinite mediating and moderating variables [49]. A rigorous and determined program of research is required in order to respond to the request for explicit and in-depth theories of change. However, whilst developing and refining theoretical frameworks for each specific discipline within outdoor therapies practice, we may collectively, as a field, join forces to arrive at an overall ontology of interconnectedness [50] as a metatheory for outdoor healthcare rather than striving to identify singular explanations tying therapeutic process and nature together in causal explanations. Instead, accept that our presence IN nature is that of an embodied experience AS nature—where both human and more than human are simply parts of a whole. As an analogy, should we be seeking the causal link between oxygen and psychotherapy? While obvious biological and physiological arguments can be made for the benefits of effective breathing and clean air, maybe the reasons are too evident and unnecessary to formulate via research for therapeutic outcomes? In a coherent system approach, humans play an equal part in human flourishing and in the relationality between humans and nature, and further remind us of the false dichotomy of separation between the two [51]. In this regard, a holistic theory of integrated relatedness may be developed as a parallel expression of support for outdoor therapies while the explanatory science catches up.

## Figures and Tables

**Table 1:** Search protocols by field and subject terms, and studies located by database.

Search Number	Field	Search Terms	Database Results
S1	SU (Subjects)	therapy or counselling or counseling or psychotherapy	PsychInfo 374,657 Medline 3,321,539 Academic S.P. 340,105
S2	SU (Subjects)	AND wilderness OR adventure OR nature* OR outdoor OR forest OR garden OR animal-assisted OR equine OR horticultural OR green* OR blue*	PsychInfo 1865 Medline 9883 Academic S.P. 1611
S3	Open	AND “systematic review” OR “meta-analysis”	PsychInfo 54 Medline 107 Academic S.P. 42

**Table 2:** PICOS modified statement of eligibility for inclusion.

	Criteria	As Defined in the Present Review
**P**	Population(s)	Health professionals and clients/participants, no age discrimination
**I**	Phenomena of interest/ intervention	Intentionally planned outdoor and nature-based interventions/ therapy for change, growth, development, and healing
**C**	Context	Therapy in/with natural environments
**O**	Outcome/Endpoint	An expression of nature’s role in outdoor therapy (as possible theory or mechanism of change/processes)
**S**	Study design	Systematic reviews and meta-analyses only

**Table 3:** Characteristics of included studies.

Author/Year	Population	Intervention	Treatment Goals	Study Design
[27] Annerstedt & Währborg, 2011	Youth and adults	Nature-based therapy	Addressing states of ill health, mental disorders, substance abuse or addiction, dementia, and varied physical disorders	Systematic review
[28] Bettmann et al., 2016	Youth	Wilderness therapy	Addressing substance abuse or addiction, mental health and behavioral issues	Meta-analysis
[18] Bowen & Neill, 2013	Youth and adults	Adventure therapy	Addressing substance abuse or addiction, mental health issues, behavioral and interpersonal problems	Meta-analysis
[29] Britton et al., 2020	Youth and adults	Nature-based therapy (blue space)	Improving psychological, social and/or physical wellbeing	Systematic review
[30] Corazon et al., 2019	Adults	Nature-based therapy	Seeking stress recovery	Systematic review
[31] Djernis et al., 2019	Youth and adults	Nature-based therapy (mindfulness)	Improving psychological, physiological and/or interpersonal outcomes	Systematic review & meta-analysis
[32] Gillis et al., 2016	Youth	Wilderness therapy	Addressing substance abuse or addiction, mental health and behavioral issues	Meta-analysis
[33] Kamioka et al., 2014	Youth and adults	Horticultural therapy	Addressing mental and physical disorders	Systematic review
[34] Kotera et al., 2020	Youth and adults	Forest (and nature-based) therapy	Addressing depression, anxiety, stress and anger	Systematic review & meta-analysis
[35] Lee et al., 2017	Adults	Forest therapy	Addressing depression	Systematic review
[36] Mygind et al., 2019	Children and youth	Nature-based therapy	Addressing mental, physical and social health	Systematic review
[37] Nicholas et al., 2019	Older adults (60+)	Horticultural therapy	Addressing physical and cognitive decline	Systematic review
[38] Wen et al., 2019	Open	Forest therapy	Addressing health effects	Systematic review
[39] Zhao et al., 2020	Adults	Horticultural therapy	Addressing dementia	Systematic review
